# Pemphigus vulgaris: a case report

**DOI:** 10.11604/pamj.2022.42.184.34184

**Published:** 2022-07-07

**Authors:** Janumpally Varshitha Thanmai, Gantala Ramlal, Katne Tejaswi, Inukonda Lakshmi Mounica

**Affiliations:** 1 Department of Oral Medicine and Radiology, SVS Institute of Dental sciences, Mahabubnagar- 509002, Telangana, India

**Keywords:** Pemphigus vulgaris, autoimmune disorder, acantholysis, corticosteroids, case report

## Abstract

Pemphigus vulgaris (PV) is an autoimmune mucocutaneous disorder of the oral cavity and is the most common subtype of pemphigus. The etiology remains obscure, although the disease is characterized by autoantibodies directed against the desmoglein component of the keratinocytes. It manifests clinically as vesicle, bullae or erosions of skin and mucous membrane and histopathologically shows the presence of acantholysis. The presence of exclusive oral lesions initially increases the chances of misdiagnosing the disease as another condition, posing diagnostic, therapeutic and prognostic difficulties, consequently prompt diagnosis and treatment can prevent untoward consequences. Demonstration of IgG antibodies against desmoglein in Immunofluroscence confirms the diagnosis. In here we report a case of a 55-year-old female patient suffering from PV emphasizing the significance of clinical examination, pertinent investigations, treatment rendered and its outcome.

## Introduction

Wichman in 1791 coined the term “pemphigus” which is derived from the Greek word pemphix, denoting a blister or bubble, referring to a group of autoimmune, mucocutaneous blistering diseases [1]. It is an uncommon disease with a global incidence ranging from 0.5 to 3.2% per million population per year and the prevalence varies as per the geographic area and ethnic population. In India, the prevalence is around 0.09% to 1.8% which is lesser than the rest of the world. This disease occur with a greater prevalence amongst women, occurring at a rate of 2 to 1 and shows a peak in the fifth and sixth decade of life [2].

The variants of pemphigus include pemphigus vulgaris (PV), pemphigus vegetans, pemphigus foliaceous, pemphigus erythematosus, paraneoplastic pemphigus (PNP), drug-related pemphigus, immunoglobulin A (IgA) pemphigus. Of, the two major variants are PV and pemphigus foliaceus which can be differentiated from each other by level of acantholysis, with the former in the suprabasilar level and the latter in the subcorneal level [3]. PV is the most common form of pemphigus, accounting for more than 80% of all cases with oral lesions. The oral lesions generally precede the skin lesions and can occur anywhere on the oral mucosa, the commonest site being buccal mucosa followed by palatal, lingual and labial mucosa [1]. Desquamative gingivitis is the commonest manifestation of the disease when gingival is involved [2]. Early recognition and treatment help in preventing the progression of the disease to skin involvement.

## Patient and observation

**Patient information:** a-55-year-old female patient reported to the Department of Oral Medicine and Radiology Outpatient Department (OPD) with a chief complaint of extensive painful ulcers in the mouth since 3 months with dysphagia and a burning sensation on intake of hot and spicy food.

**Figure 1:**
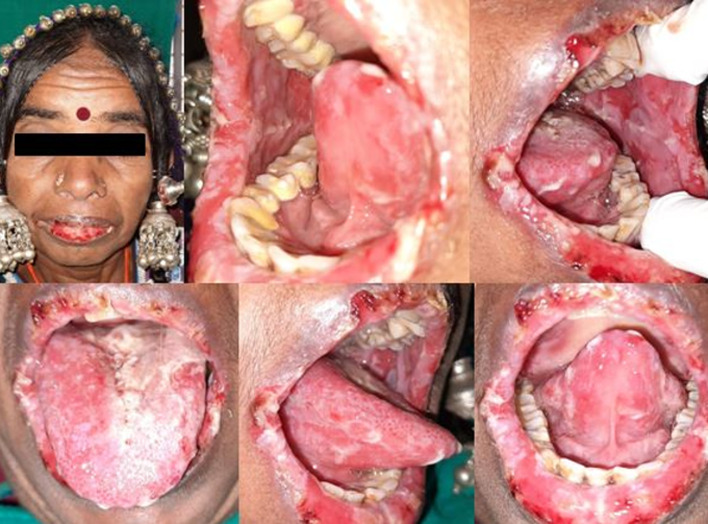
series of pictures showing irregular ulcerations and encrustations on lips and upper and lower labial mucosa, bilateral buccal mucosa, dorsal and ventral surfaces of the tongue

**Clinical findings:** on clinical examination, irregular ulcerations and encrustations were noticed on the lips and diffuse erosions and ulcerations of variable sizes and irregular borders with peripheral oedema were noticed on the upper and lower labial mucosa, bilateral buccal mucosa, dorsal and ventral surfaces of the tongue (Figure 1). Peripheral extension of the lesion was noticed on applying tangential pressure to the lesion indicating a positive nikolysky sign. A provisional diagnosis of PV was made considering the above findings.

**Figure 2:**
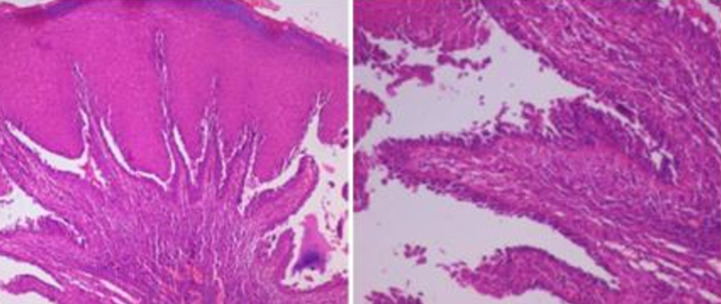
H and E sections showing hyperplastic parakeratinized stratified squamous epithelium with broad elongated retepegs, the superficial epithelium shows intercellular edema, ulceration with suprabasillar split, and separation of parabasal layer of the epithelium

**Diagnostic assessment:** haematological and biochemical investigations were carried out, autoantibodies against desmoglein 1 and 3 were detected in indirect immunofluorescence and an incisional biopsy was performed from peri lesional site of the right buccal mucosa and was sent for histopathological (Figure 2).

**Diagnosis and therapeutic interventions:** a definitive diagnosis of PV was made and initially, the patient was prescribed prednisolone tablets 20 mg, which were to be taken thrice daily along with vitamin supplements along with the topical application of triamcinolone gel, 0.1% twice daily on the mucosal lesions.

**Figure 3:**
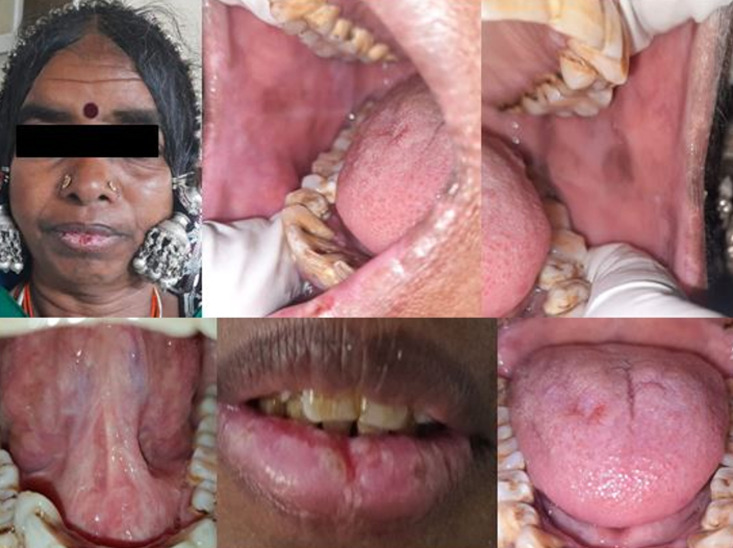
complete regression of lesions on lips and upper and lower labial mucosa, bilateral buccal mucosa, dorsal and ventral surfaces of the tongue

**Follow-up and outcome of interventions:** on the first follow-up gradual reduction of the areas of ulcerations was seen and the oral hygiene of the patient was monitored and scaling was done. On the second follow-up, marked improvement of the lesions is noticed and the dose was tapered to 40 mg (Figure 3). Over the past 6 months, prednisolone was gradually reduced and the patient is on remedial therapy of prednisolone 5 mg, and so far no other lesions have been reported.

**Informed consent:** written informed consent was obtained from the patient.

## Discussion

Pemphigus is a life-threatening mucocutaneous disorder affecting the skin and mucous membrane and the classic presentation is a thin-walled bulla arising from otherwise normal skin or mucosa, which breaks rapidly and extends peripherally, leaving large denuded areas [3]. This disease exhibits a characteristic “Nikolsky´s sign” which is the ability to induce peripheral extension of a blister and/or sloughing of epidermis as a consequence of exercising oblique pressure with a finger or thumb to the affected, peri lesional or normal skin [1-4]. The positive Nikolsky´s sign in the present case gave us a clue to the diagnosis of the disease.

The “Desmoglein compensation theory” and “Multiple hits hypothesis” are among the several other proposed concepts explaining the pathophysiology of Pemphigus, cognated by the mechanism of primary acantholysis [5]. The intercellular adhesion proteins, desmosomes contribute to the epidermal cell-cell junction by anchoring the keratin intermediate filament to the cell membrane of epidermal cells. Desmosomal proteins, desmoglein 1 (DSG 1- major component of epidermal desmosomes) and desmoglein 3 (DSG 3- cadherin or adhesion protein in oral mucosa) plays a role in skin adhesion. In PV IgG autoantibodies bind with desmosomal transmembrane adhesion molecules, desmocollin, non-desmosomal proteins (such as pemphaxin, alpha 9-acetylcholine receptor and thyroperoxidase) and mitochondrial proteins resulting in primary acantholysis [2]. Patients with certain HLA genotypes (particularly DR4), concomitant factors such as drugs (penicillin, cephalosporin, captopril, aspirin, levodopa, heroin), viruses (herpes virus), foreign substances (organophosphate exposure, allergens), radiation, anxiety, stress, and diet are predisposed to pemphigus.

In 70-90% of the cases of PV oral mucosa is involved prior to the involvement of the skin. The oral manifestations of PV typically run a chronic course causing blisters, erosions and ulcers of the oral mucosa with the commonest sites being the areas subjected to frictional trauma like buccal mucosa, lips, and soft and hard palate, gingival and may affect other membranes including the conjunctiva, nasal mucosa, pharynx, larynx, esophagus and genital mucosa. The subjective symptoms include increased salivation, problems with chewing and swallowing, burning sensation [3]. The oral lesions are the intial clinical presentation of PV which can be accompanied by cutaneous manifestation. Healing occurs slowly, with no scar formation.

Investigations involve biopsy, direct immunofluorescence (DIF), indirect immunofluorescence (IDIF) and detection of desmoglein 1 and 3. Biopsy for PV is done best when the vesicles and bulla are intact and are less than 24 hour old, preferably from the advancing edge of the lesion. Supra-basilar acantholysis with basal cells appearing as a row of “tomb-stone” seen in PV helps differentiate from other diseases like mucous membrane pemphigoid, bullous lichen planus and chronic ulcerative stomatitis which are subepithelial blistering diseases. A second biopsy is to be studied by DIF for PV, where the test will demonstrate the presence of IgG in conjunction with complement component 3, IgA and IgM, in a “chicken wire/fish-net” appearance, in the intercellular spaces of affected oral epithelium. The autoantibodies that are directed against Dsg-1 and Dsg-3 can be identified in the circulating blood serum using an IDIF assay that employs oldworld monkey esophagus as a target substrate. IDIF is less sensitive than DIF and can be considered if biopsy is difficult. DSG 1 and 3 in serum sample of PV patients is usually detected by ELISA [5].

Early diagnosis is vital for patient management, where lower doses for shorter periods can effectively control the disease. And the main purpose of the treatment is to control the disease progression and prevention of relapse. The systemic and topical corticosteroids are considered as gold standard for the treatment of PV in combination with immunosuppressive steroid-sparing agents. New remedies such as intravenous immunoglobulins which has immunomodulatory action and targeted therapy are being considered as treatment options for pemphigus markedly than corticosteroids which have immunosuppressive actions [6]. If left untreatment, this condition can be fatal with a mortality rate ranging from 50% to 100% because of extensive skin involvement, loss of the epidermal barrier function leading to electrolyte imbalance and secondary bacterial and systemic infection, bronchopneumonia. Institution of early treatment could prevent such serious complications [7].

## Conclusion

PV is an immune-mediated disorder with increased tendency to affect the oral mucosa, and if left untreated it could lead to patient´s death. Early disease probably is easier to control than widespread disease Therefore timely diagnosis and prompt treatment plan should be outlined to increase patients´ quality of life, and should be directed to accelerate remission, minimize flare-ups, hospitalization, and morbidity associated with therapeutic agents.
